# Peer navigation to support psychosocial well-being and HIV treatment among adolescents: A rapid review

**DOI:** 10.4102/hsag.v31i0.3464

**Published:** 2026-09-29

**Authors:** Charné Petinger, Bernard N. Mabuza, Brian van Wyk

**Affiliations:** 1 School of Public Health, Faculty of Community and Health Sciences, University of the Western Cape, Cape Town, South Africa

**Keywords:** HIV, ALHIV, peer navigation, peer support, sub-Saharan Africa, review

## Abstract

**Background:**

Adolescents living with HIV (ALHIV) in sub-Saharan Africa experience poorer outcomes across the HIV care cascade than other age groups because of intersecting developmental, psychosocial and structural barriers. Peer navigation has emerged as a promising strategy to support engagement in care, yet evidence on its effectiveness and implementation for ALHIV remains fragmented.

**Aim:**

To synthesise evidence on peer navigation and peer-led psychosocial support interventions for adolescents living with HIV.

**Method:**

A rapid review was conducted in accordance with PRISMA 2020 principles. PubMed and EBSCOhost databases were searched for qualitative, quantitative and mixed-methods studies published between 2013 and 2025.

**Results:**

Fourteen studies conducted across eight countries were included. Peer navigation interventions were typically delivered by young adults living with HIV who were stable on treatment and trained to provide psychosocial, adherence and health system navigation support. Most studies reported improvements in antiretroviral therapy adherence, retention in care and viral suppression, although effects varied by context and were not always sustained. Psychosocial benefits included reduced stigma, strengthened trust and improved self-efficacy. Implementation findings indicated high acceptability and feasibility, supported by structured training and supervision, alongside challenges related to sustainability and structural barriers such as transportation costs.

**Conclusion:**

Peer navigation is a valuable component of adolescent-friendly HIV care in sub-Saharan Africa, particularly when integrated within responsive clinical systems and complemented by strategies addressing structural vulnerability.

**Contribution:**

This review consolidates regional evidence on peer navigation for ALHIV and provides implementation-relevant insights to inform the integration and scale-up of peer-led models within public sector HIV services.

## Introduction

Adolescence and young adulthood constitute a critical developmental period characterised by rapid biological, cognitive, psychological and social change. For adolescents living with HIV (ALHIV), typically defined as individuals aged 10–24 years, these normative transitions unfold alongside the lifelong demands of antiretroviral therapy (ART), often amplifying vulnerability to disengagement from care and suboptimal treatment outcomes (Chew et al. 2025; Msosa et al. 2025; Slogrove & Sohn 2018). Managing HIV during this stage requires adolescents to balance emerging autonomy, identity formation and peer belonging with consistent treatment adherence and engagement in health services, a tension that is frequently difficult to sustain (Weyers, Crowley & Tokwe 2025).

Sub-Saharan Africa bears the largest global burden of ALHIV, with South Africa alone home to an estimated 320 000 adolescents aged 10–19 years living with HIV, representing one of the highest national adolescent HIV burdens worldwide (Joint United Nations Programme on HIV/AIDS [UNAIDS] 2024). Despite substantial progress in ART availability and paediatric HIV survival, adolescents in the region continue to experience poorer outcomes across the HIV care cascade compared to younger children and adults, including lower rates of retention in care and viral suppression (Haghighat et al. 2021; Munyayi & Van Wyk 2024; United Nations Children’s Fund [UNICEF] 2023). These disparities place adolescents at heightened risk of morbidity, mortality and the development of drug resistance during a period that has lasting implications for long-term health trajectories (Ndhlovu et al. 2021).

Poorer outcomes among ALHIV are driven by a complex interplay of developmental, structural, clinical and psychosocial factors. Adolescents must navigate normal physiological and emotional changes while managing HIV-related stigma, social isolation and challenges around disclosure to peers, schools and romantic partners (Mayman & Van Wyk 2025; Mwangwa et al. 2025). Because social acceptance is particularly salient during adolescence, fear of inadvertent disclosure often leads to treatment concealment, missed doses or disengagement from services in order to avoid being identified as living with HIV (Kiirya et al. 2025; Seunanden, Ngwenya & Seeley 2025). This period is further complicated by shifting power dynamics as adolescents seek greater independence from caregivers, which can disrupt established treatment routines and accountability structures (Balogun et al. 2025; Hung & Wei 2025).

Health system factors frequently compound these challenges. Many adolescents receive care in adult-oriented clinics that are not developmentally responsive or adolescent friendly, while clinic schedules may conflict with school or work commitments (Munyayi & Van Wyk 2024; Sherr et al. 2018). The transition from paediatric to adult HIV services, often marked by reduced psychosocial support and increased expectations of self-management, is a particularly vulnerable period associated with disengagement from care and virologic rebound (Hung & Wei 2025; Zanoni et al. 2019). Broader social determinants of health, including poverty, gender inequality, unstable housing and limited access to youth-friendly services, further exacerbate these risks in many low- and middle-income country settings (Toth et al. 2018).

Peer navigation has increasingly been recognised as a promising strategy to address these multifaceted barriers and support adolescent engagement in HIV care. Peer navigators, often young people living with HIV themselves, are uniquely positioned to provide credible, relatable and non-judgemental support grounded in shared lived experience. Peer-led interventions have been shown to improve ART adherence, retention in care and viral suppression by fostering trust, providing psychosocial support and addressing practical barriers such as forgetfulness and treatment fatigue through reminders and accompaniment (Adhiambo et al. 2025; Atujuna et al. 2021; Kwena et al. 2024; Sannigrahi et al. 2025; Zamudio-Haas et al. 2024). Peer navigators can also serve as critical bridges between communities and health systems, facilitating linkage to care, referrals and sustained engagement for adolescents who are marginalised or at high risk of disengagement (Bakari et al. 2025; Shah et al. 2019).

Beyond clinical outcomes, peer navigation plays a central role in supporting adolescents’ psychosocial well-being. Peer-led support can strengthen resilience, promote healthy disclosure decision-making, reduce internalised stigma and support adolescents through the transition from paediatric to adult care (Ahmed et al. 2022; Hung & Wei 2025). Emerging evidence also suggests that peer interventions may improve mental health outcomes, including reductions in symptoms of depression and anxiety, by providing safe spaces for empathetic discussion and problem-solving (Adjorlolo et al. 2025; Simms et al. 2022). Importantly, peer navigation models may generate reciprocal benefits, with peer mentors themselves reporting improved self-adherence, enhanced self-worth and the development of positive leadership identities within their communities (Bernays et al. 2020; Burke et al. 2022).

While evidence from adult populations in sub-Saharan Africa supports the effectiveness of peer-led approaches, the evidence base specific to adolescents remains fragmented, with substantial variation in intervention design, implementation models and outcomes. Given the distinct developmental and contextual needs of ALHIV, there is a need for focused synthesis to inform the design and scale-up of adolescent-responsive peer navigation models.

Given the increasing programmatic interest in peer navigation and peer-led psychosocial support for ALHIV, a timely synthesis of the available evidence is required to inform adolescent HIV service delivery in sub-Saharan Africa. A full systematic review may not always be feasible where evidence is needed to guide emerging implementation decisions, particularly when the aim is to identify intervention characteristics, implementation experiences and reported outcomes rather than to generate a pooled effect estimate. Rapid reviews are recognised as a form of knowledge synthesis that adapts selected components of the systematic review process to produce evidence within shorter timeframes while retaining transparent and structured methods to minimise bias (Haby et al. 2016; Smela et al. 2023; Tricco et al. 2015). This approach was therefore appropriate for the present review, as the evidence on peer navigation and peer-led models for ALHIV is dispersed across different countries, intervention designs and implementation contexts. By using a focused review question, predefined eligibility criteria and narrative synthesis, this rapid review provides a timely and policy-relevant overview of how peer-led approaches have been used to support adherence, retention in care, psychosocial well-being and treatment engagement among ALHIV in sub-Saharan African countries. This strengthens the aim of this study to inform the integration of peer navigation into adolescent-friendly HIV care packages and strengthen responses across the adolescent HIV care continuum.

## Methods

### Review design

We conducted a rapid review to synthesise evidence on the use of peer navigators and other peer-led psychosocial support interventions for ALHIV in sub-Saharan Africa. A rapid review approach was selected to allow for timely synthesis of a growing and heterogeneous evidence base while retaining key elements of systematic review methodology to ensure transparency and rigour. The review was guided by the principles outlined in the Preferred Reporting Items for Systematic Reviews and Meta-Analyses (PRISMA) 2020 statement, with methodological adaptations appropriate for rapid evidence synthesis (Page et al. 2021). These adaptations included restricting searches to selected electronic databases, limiting inclusion to studies published between 2013 and 2025 and undertaking narrative rather than quantitative syntheses because of heterogeneity in study designs, intervention models and outcome measures. The study selection process is summarised in a PRISMA flow diagram (Figure 1).

**FIGURE 1:**
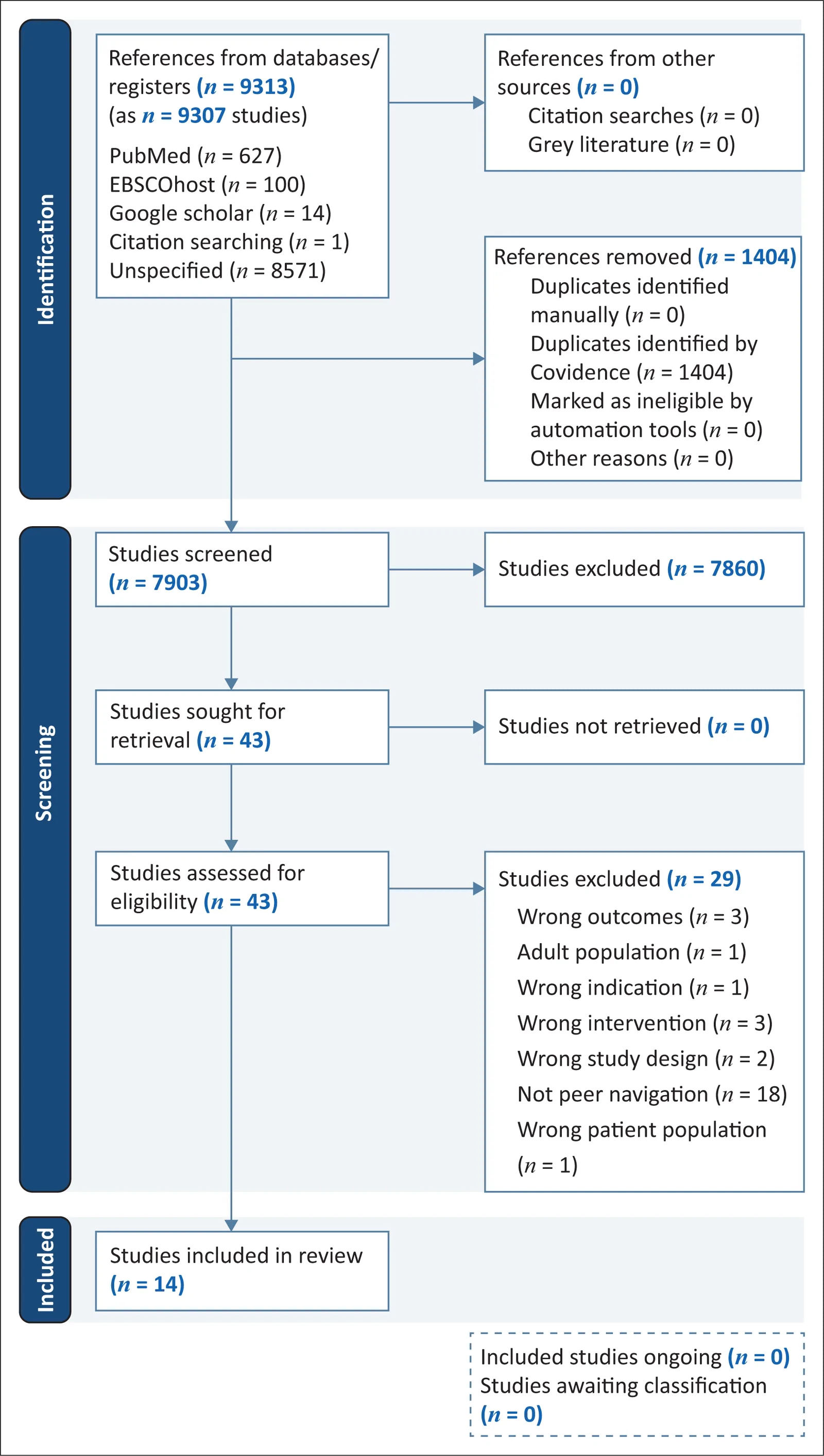
Drawn from *covidence*.

### Eligibility criteria

Eligibility criteria were defined a priori using the Population, Intervention, Comparison, Outcome, Timeframe (PICOT) framework. Population, Intervention, Comparison, Outcome, Timeframe was selected as it provides a systematic approach to defining review questions and inclusion criteria, particularly for reviews evaluating the effectiveness and implementation of health interventions across heterogeneous study designs.

- *Population*: Adolescents living with HIV aged 10–19 years.
- *Intervention*: Any intervention delivering peer navigation or peer-led psychosocial support specifically targeted at adolescents living with HIV.
- *Comparison*: Studies with or without a comparison group were eligible for inclusion.
- *Outcomes*: Any health-related outcomes, including but not limited to ART adherence, self-efficacy, engagement or retention in care, mental well-being and quality of life.
- *Timeframe*: Studies published between 2013 and 2025.
- *Types of studies*: Qualitative, quantitative and mixed-methods primary research studies.

Studies were excluded if they were conducted outside sub-Saharan Africa. Sub-Saharan Africa was selected as the geographic focus because it remains the region most affected by adolescent HIV and is therefore a priority context for interventions that strengthen adherence, retention in care and psychosocial support among ALHIV (UNAIDS 2024). Within the high-burden and resource-constrained settings in sub-Saharan Africa, peer navigation and peer-led psychosocial support are increasingly used as adolescent-responsive strategies across the HIV care continuum (Ahmed et al. 2022; Mark et al. 2019). Studies were also excluded if they did not include adolescents as a clearly defined population or did not report primary empirical findings, such as editorials, commentaries or study protocols without results. These criteria were applied to ensure the review remained focused on evidence directly relevant to ALHIV and was informed by primary data on intervention outcomes and implementation experiences, thereby strengthening the validity and applicability of the review findings.

### Search strategy

The search strategy was initially developed in PubMed to identify relevant Medical Subject Headings and optimise sensitivity and specificity. It was refined in accordance with the Peer Review of Electronic Search Strategies guidelines to ensure methodological robustness. The final search was conducted across PubMed and EBSCOhost databases, including Cumulative Index to Nursing and Allied Health Literature (CINAHL), Academic Search Complete and American Psychological Association (APA) PsycArticles.

Search terms combined concepts related to adolescence, HIV, peer navigation or peer support and sub-Saharan Africa. Database-specific adaptations were applied to account for differences in indexing and search functionality. Reference lists of included studies and relevant review articles were manually screened to identify additional eligible publications. The final search was conducted on 22 July 2025. The full PubMed search strategy is provided in Box 1.

BOX 1 PubMed search strategy.

((((((Peer navigation) OR (Peer support)) AND (Youth)) OR (Adolescent)) OR (ALHIV)) AND (HIV)) OR (AIDS) AND (Sub-Saharan Africa)

### Study selection

All identified records were imported into covidence systematic review software, and duplicate citations were removed. Titles and abstracts were independently screened by two reviewers against the predefined eligibility criteria. Full-text articles were subsequently assessed for inclusion by the same reviewers. Screening decisions were subsequently compared and discussed to ensure consistent application of the inclusion and exclusion criteria. Where clarification was required regarding study eligibility, the senior reviewer was consulted to verify inclusion decisions and confirm the final set of studies included in the review.

### Data extraction

A standardised data extraction template was developed and piloted within covidence to ensure consistency and completeness. Extracted information included author and year of publication, country and setting, study design, participant characteristics and sample size, description of the peer navigation or peer-led intervention, mode of delivery, underlying theoretical or conceptual framework where reported, outcomes measured and key findings. One reviewer conducted the data extraction, and a second reviewer independently checked the extracted data for accuracy and completeness.

### Data synthesis

A narrative synthesis approach was used to summarise and integrate findings across studies. This approach was selected because of substantial heterogeneity in study designs, intervention models, populations and outcome measures. Findings were organised into three summary tables: (1) characteristics of included studies, (2) descriptions of peer navigation and peer-led interventions and (3) summaries of primary, secondary and implementation-related outcomes.

Where available, both quantitative and qualitative findings were synthesised to provide a comprehensive understanding of intervention effectiveness, acceptability and implementation considerations. Meta-analysis was not undertaken as a result of methodological and clinical heterogeneity across studies.

### Ethical considerations

Ethical clearance to conduct this study was obtained from the Biomedical Research Ethics Committee of the University of the Western Cape (No. BM25/1/16).

## Review findings

A total of 14 studies met the inclusion criteria for this rapid review, representing peer-led interventions implemented across eight countries in sub-Saharan Africa: South Africa, Kenya, Tanzania, Nigeria, Zimbabwe, Uganda, Eswatini and Malawi. Most studies were conducted in public sector ART clinics or community-based health settings and targeted ALHIV. Study designs included quasi-experimental pre–post evaluations, mixed-methods pilot or feasibility studies and qualitative studies exploring implementation experiences. Sample sizes varied substantially, ranging from small qualitative cohorts of approximately 15 participants to larger quantitative cohorts exceeding 60 participants, with most study populations comprising adolescents and young adults aged 15–24 years. The characteristics of included studies are summarised in Online Appendix 1, Table 1-A1.

As shown in Online Appendix 1, Table 1-A1, studies were implemented across diverse geographic and health system contexts, with a concentration in Nigeria, Kenya, Tanzania and South Africa. Recruitment was predominantly clinic based, with participants identified through ART registers and approached by study staff or peer navigators. Across studies, peer-led interventions were embedded within routine service delivery or pilot programmes and consistently involved peers living with HIV who provided psychosocial, adherence and navigation support. Despite variation in intervention structure, intensity and duration, most studies reported improvements in at least one clinical or psychosocial outcome following implementation.

Online Appendix 1, Table 2-A1 summarises the characteristics of peer navigation interventions. Peer navigators were typically young adults living with HIV who were virally suppressed and actively engaged in care, enabling them to serve as credible and relatable role models. In several studies, peer navigators were intentionally matched to participants by age, gender or shared lived experiences to strengthen rapport and trust. Training processes were generally structured and comprehensive, covering topics such as adolescent-friendly health services, ART adherence support, confidentiality, motivational interviewing and health system navigation. Many programmes incorporated role-playing, refresher training and ongoing supervision to support fidelity and navigator well-being.

Intervention activities commonly included individual or group-based adherence counselling, appointment reminders, accompaniment to clinic visits and emotional support. Several interventions extended beyond clinic-focused support to include social and structural navigation, such as facilitating referrals to mental health services, social support programmes or community-based resources. Delivery was primarily in-person, often within clinic settings, but many studies reported the use of hybrid approaches that incorporated phone calls or text messaging to maintain contact and provide flexible support. Theoretical underpinnings were most frequently informed by social support theory, adolescent-friendly health service principles or broader behaviour change frameworks.

Online Appendix 1, Table 3-A1 presents a synthesis of primary, secondary and implementation-related outcomes. The most commonly reported primary outcomes were viral suppression, ART adherence and retention or engagement in care, with the majority of studies demonstrating improvements following intervention implementation. Secondary outcomes included psychosocial well-being, self-efficacy, health literacy and perceived social support, which also showed positive changes in most studies. Qualitative findings consistently emphasised the importance of trust, shared lived experience and the non-judgemental support provided by peer navigators in reducing stigma and strengthening engagement with HIV care.

Implementation-related findings indicated high acceptability and perceived feasibility of peer navigation interventions among adolescents, peer navigators and healthcare providers. However, studies also identified persistent challenges, including sustaining funding, ensuring consistent supervision and support for peer navigators and addressing structural barriers such as transportation costs and competing school or work commitments. These findings highlight both the promise of peer-led approaches and the need for deliberate planning to support long-term integration and sustainability within routine adolescent HIV care.

## Discussion

This rapid review synthesised evidence from 14 studies examining peer navigation and peer-led psychosocial support interventions for ALHIV in sub-Saharan Africa. Overall, the findings suggest that peer navigation is a highly acceptable and feasible strategy that can improve engagement across the HIV care cascade, particularly ART adherence, retention in care and psychosocial well-being. Across diverse contexts, peer-led interventions consistently leveraged shared lived experience to build trust, reduce stigma and strengthen adolescents’ capacity to remain engaged in care (Adhiambo et al. 2025; Ahmed et al. 2022; Bernays et al. 2020; Burke et al. 2022).

Several studies reported improvements in clinical outcomes, including viral suppression and retention in care although effects were not uniform across settings or sustained in all populations. Interventions reporting stronger gains in viral suppression typically combined peer navigation with structured adherence support, frequent contact and integration within routine clinic services (Ahonkhai et al. 2023; Balogun et al. 2025; Garofalo et al. 2022; Kopo et al. 2023). In contrast, studies among adolescents with advanced treatment failure or high baseline drug resistance demonstrated only transient improvements in viral outcomes (Ndhlovu et al. 2021). These findings highlight the limits of peer support when not accompanied by responsive clinical management, including timely regimen switching and access to resistance-informed treatment, and reinforce that peer navigation is most effective when embedded within functioning health systems rather than implemented as a standalone intervention.

Beyond clinical outcomes, peer-led interventions demonstrated consistent psychosocial benefits. Qualitative findings across studies highlighted trust, confidentiality and shared lived experience as central mechanisms through which peer navigation reduced internalised stigma, social isolation and fear of disclosure (Ahmed et al. 2022; Bernays et al. 2020; Burke et al. 2022). Adolescents reported that peer navigators created safe, non-judgemental spaces that enabled open discussion of adherence challenges and supported decision-making around disclosure and care engagement (Adhiambo et al. 2025; Hosaka et al. 2021). Several interventions also reported improvements in self-efficacy, health literacy and resilience, outcomes that are particularly salient during adolescence and may contribute to sustained engagement even when short-term viral outcomes are modest (Hosaka et al. 2021; Simms et al. 2022).

Implementation findings across studies identified several core features underpinning successful peer navigation models. Peer navigators were typically young adults living with HIV who were virally suppressed and stable in care, reinforcing their credibility as role models (Ahmed et al. 2022; Bernays et al. 2020). Structured training, ongoing supervision and clear role delineation were repeatedly emphasised as essential for maintaining intervention quality and supporting peer navigators in managing the emotional labour associated with their roles (Burke et al. 2022; Wambua et al. 2019). Interventions that incorporated flexible delivery modalities, including phone-based or hybrid models, were perceived as particularly acceptable by adolescents, enabling continuity of support while accommodating school schedules, stigma concerns and transportation barriers (Adhiambo et al. 2025; Ahonkhai et al. 2023).

Despite high acceptability, persistent structural constraints limited the reach and sustainability of peer-led interventions. Transportation costs, food insecurity, inconsistent access to mobile data and competing educational or economic demands were commonly reported barriers that undermined consistent engagement, particularly for adolescents in rural or underserved settings (Balogun et al. 2025; Shah et al. 2019). These findings align with broader evidence that adolescent HIV outcomes are shaped by social and economic vulnerability and suggest that peer navigation should be integrated with differentiated service delivery approaches, including community-based ART delivery and multi-month dispensing, to address these structural determinants.

Emerging evidence also points to the adaptability of peer navigation within evolving models of HIV care. Studies exploring digital peer navigation and the potential role of peer mentors in the rollout of long-acting injectable ART highlight opportunities for peer-led approaches to support treatment literacy, linkage to care and sustained engagement as service delivery models shift (Adhiambo et al. 2025; Bakari et al. 2025). However, successful scale-up will require formal recognition of peer navigator roles within health systems, including standardised training, remuneration and career pathways, to ensure ethical implementation and long-term sustainability (Bakari et al. 2025; Bernays et al. 2020).

Taken together, the findings of this rapid review indicate that peer navigation constitutes a critical component of adolescent-friendly HIV care in sub-Saharan Africa. Its greatest impact is observed when psychosocial support is integrated within responsive clinical systems and complemented by strategies that address structural vulnerability. Future research should focus on clarifying mechanisms of effect across different adolescent subgroups, evaluating long-term outcomes and examining how peer navigation can be optimally integrated into differentiated and emerging HIV service delivery models.

### Strengths and limitations of this study

This rapid review has several strengths. Firstly, it synthesises evidence across a diverse range of peer navigation and peer-led psychosocial support interventions for adolescents and young adults living with HIV in sub-Saharan Africa, capturing clinical, psychosocial and implementation-related outcomes. By including qualitative, quantitative and mixed-methods studies, the review provides a comprehensive understanding of not only whether peer navigation interventions are effective but also how and under what conditions they are implemented and experienced. This breadth is particularly valuable in the context of adolescent HIV care, where outcomes are shaped by complex developmental and social factors.

Secondly, the review adopts a structured and transparent methodological approach, guided by PRISMA 2020 principles and a clearly defined PICOT framework. The use of independent screening, standardised data extraction and narrative synthesis enhances the credibility and reproducibility of the findings. Additionally, the focus on peer navigation models implemented within routine health services and community settings strengthens the relevance of the findings for policy and practice in resource-constrained contexts.

Several limitations should be considered when interpreting the findings. As a rapid review, methodological streamlining may have resulted in the omission of some relevant studies, particularly unpublished literature or studies indexed outside the selected databases. The heterogeneity of study designs, intervention models, populations and outcome measures limited the ability to conduct meta-analysis and made direct comparison across studies challenging. Many included studies were pilot or feasibility evaluations with small sample sizes and short follow-up periods, which restricts the strength of causal inference and the assessment of long-term outcomes.

In addition, several studies relied on self-reported measures of adherence and engagement in care, which are subject to recall and social desirability bias. The majority of studies were conducted in clinic-based populations, potentially underrepresenting adolescents who are disengaged from care or face the most severe structural barriers. Finally, reporting on implementation fidelity, costs and sustainability was variable across studies, limiting the ability to draw firm conclusions regarding scalability and integration within health systems.

## Conclusion

This rapid review provides evidence that peer navigation and peer-led psychosocial support interventions are acceptable, feasible and potentially effective strategies for improving engagement in HIV care among adolescents and young adults living with HIV in sub-Saharan Africa. Across diverse settings, peer navigation consistently demonstrated benefits for ART adherence, retention in care and psychosocial well-being by leveraging shared lived experience to reduce stigma, build trust and support adolescents through critical developmental and health system transitions.

However, the findings also indicate that peer navigation alone is insufficient to address all drivers of poor clinical outcomes, particularly in the context of advanced treatment failure, drug resistance and persistent structural vulnerabilities. The greatest impact of peer-led interventions appears to occur when they are integrated within responsive clinical systems and complemented by differentiated service delivery models and broader social support mechanisms.

As HIV care continues to evolve, including the expansion of digital health platforms and long-acting ART, peer navigation offers a flexible and adaptable approach to supporting adolescents across the care continuum. Strengthening peer navigation programmes will require intentional investment in training, supervision, remuneration and formal integration within health systems. Future research should prioritise longitudinal evaluation, implementation outcomes and equity-focused approaches to ensure that peer-led models contribute to sustained improvements in adolescent HIV outcomes and the long-term well-being of young people living with HIV.
